# Mice with a heterozygous *Lrp6* deletion have impaired fracture healing

**DOI:** 10.1038/boneres.2016.25

**Published:** 2016-09-06

**Authors:** Travis A Burgers, Juan F Vivanco, Juraj Zahatnansky, Andrew J Vander Moren, James J Mason, Bart O Williams

**Affiliations:** 1 Center for Cancer and Cell Biology, Program for Skeletal Disease and Tumor Microenvironment, Van Andel Research Institute, Grand Rapids, MI, USA; 2 Facultad de Ingenieria y Ciencias, Adolfo Ibáñez University, Viña del Mar, Chile; 3 Padnos College of Engineering and Computing, Grand Valley State University, Grand Rapids, MI, USA

## Abstract

Bone fracture non-unions, the failure of a fracture to heal, occur in 10%–20% of fractures and are a costly and debilitating clinical problem. The Wnt/β-catenin pathway is critical in bone development and fracture healing. Polymorphisms of linking low-density lipoprotein receptor-related protein 6 (LRP6), a Wnt-binding receptor, have been associated with decreased bone mineral density and fragility fractures, although this remains controversial. Mice with a homozygous deletion of *Lrp6* have severe skeletal abnormalities and are not viable, whereas mice with a heterozygous deletion have a combinatory effect with Lrp5 to decrease bone mineral density. As fracture healing closely models embryonic skeletal development, we investigated the process of fracture healing in mice heterozygous for *Lrp6* (*Lrp6* ^+/−^) and hypothesized that the heterozygous deletion of *Lrp6* would impair fracture healing. Mid-diaphyseal femur fractures were induced in *Lrp6* ^+/−^ mice and wild-type controls (*Lrp6* ^+/+^). Fractures were analyzed using micro-computed tomography (μCT) scans, biomechanical testing, and histological analysis. *Lrp6* ^+/−^ mice had significantly decreased stiffness and strength at 28 days post fracture (PF) and significantly decreased BV/TV, total density, immature bone density, and mature area within the callus on day-14 and -21 PF; they had significantly increased empty callus area at days 14 and 21 PF. Our results demonstrate that the heterozygous deletion of *Lrp6* impairs fracture healing, which suggests that *Lrp6* has a role in fracture healing.

## Introduction

The Wnt/β-catenin pathway is critical in bone development.^1,2^ The importance of this pathway to bone development was first demonstrated with a report linking low-density lipoprotein receptor-related protein 5 (LRP5) mutations and the pediatric syndrome, osteoporosis pseudoglioma.^3^ Soon after, two studies showed that point mutations in LRP5 cause increased bone mass.^4,5^ The Wnt/β-catenin pathway is initiated when a Wnt protein that binds to the complex that includes Lrp5, Lrp6, and Frizzled.^6^ Downstream, this allows β-catenin to avoid ubiquitin-dependent destruction that occurs through disheveled and glycogen synthase kinase 3. Increased levels of β-catenin cause nuclear translocation that causes TCF (transcription factor)/LEF (lymphoid enhancer-binding factor)- and TAZ (transcriptional coactivator with a PDZ-binding domain)-mediated gene transcription.^7,8^

Polymorphisms of LRP6 have been associated with decreased bone mineral density and fragility fractures,^9–12^ although others have not confirmed the results.^13–15^ Mice with a homozygous deletion of *Lrp6* have severe skeletal abnormalities and are not viable.^16^ Mice with heterozygous *Lrp6* deletion have a combinatory effect with Lrp5 to decrease bone mineral density.^17,18^

Similar to embryonic skeletal development,^19^ fracture repair utilizes regulated chondrogenic and osteoblastic phases of bone formation: first, inflammation; second, cartilaginous callus formation; third, endochondral ossification; and fourth, bone remodeling.^20^ A non-union occurs if this process is disturbed and the fracture healing lasts longer or does not complete at all. Non-unions occur in 10%–20% of fractures^21,22^ and lead to increased treatment costs and patient morbidity.^23^ The Wnt/β-catenin pathway is also important in fracture healing.^20,24–26^ Mice with a homozygous deletion of Lrp5 have impaired fracture healing,^27^ whereas mice with a homozygous deletion of secreted frizzled-related protein 1, a Wnt/β-catenin inhibitor, have improved fracture healing.^28^ To the authors’ knowledge, there have been no studies investigating the role of *Lrp6* in fracture healing.

Owing to the importance of the Wnt/β-catenin signaling pathway, specifically LRP6, on bone development and the importance of the Wnt/β-catenin signaling pathway in fracture healing, we hypothesize that the heterozygous deletion of *Lrp6* will impair fracture healing.

## Materials and methods

### Animal model and specimen preparation

This study was approved by the Institutional Animal Care and Use Committee at the Van Andel Research Institute (Grand Rapids, MI, USA). Mice on a C57BL/6J background with a heterozygous deletion of *Lrp6* (*Lrp6* ^+/−^) and wild-type control (*Lrp6* ^+/+^) mice were previously generated.^29^ To examine fracture healing and biomechanical characteristics, surgery was performed on approximately equivalent numbers of male and female mice that were 11–12 weeks old, and the right femur was fractured.

Each mouse was anesthetized using a subcutaneous weight-matched dose of tribromoethanol (average 350 μL dose of 0.079 mg·μL^−1^ solution; Avertin; Winthrop Laboratories, New York, NY, USA). A 23-gauge needle was surgically inserted in the femoral medullary canal of the right femur and a femoral fracture was created at the midshaft using a blunt impact force in a three-point bending technique, following an established procedure.^30^ Pain was managed postoperatively with subcutaneous doses of tramadol^31^ (20 mg·kg^−1^; Sigma-Aldrich, St Louis, MO, USA) administered at the time of surgery and at 12, 24, and 36 h after fracture. Mice were killed at 7, 14, 21, and 28 days post fracture (PF). Mice were divided into one group for biomechanical and micro-computed tomography (μCT) evaluation (*n*=99) and one group for histological evaluation (*n*=45). For the biomechanical and μCT evaluation, 34 fractured femurs (from 16 *Lrp6* ^+/+^ and 18 *Lrp6* ^+/−^ animals) were excluded because the fracture was oblique, comminuted, or incomplete, as determined by follow-up radiographs.^32^

### Biomechanical evaluation

After killing of each animal for biomechanical and μCT analyses, both the fractured and intact femurs were excised and cleaned of the surrounding soft tissue. The intramedullary needle in the fractured femur was removed and samples were stored at −20 °C in saline-saturated gauze.

For biomechanical assessment of the bones, all femurs were removed from the freezer, rehydrated in saline, and allowed to equilibrate to room temperature. Four-point bending mechanical testing was performed on the right femur according to the same procedure as our previous work.^32^ Force and displacement were directly measured using the TestResources (TestResources, Shakopee, MN, USA) system. Stiffness (defined as the ratio between force and displacement in the representative linear region) and maximum strength (maximum load) were calculated in Excel (Microsoft, Redmond, WA, USA). The coefficient of determination (*R* ^2^) of the stiffness measure was 0.998 9±0.001 5 (average±s.d.). The stiffness ratio and maximum load ratio were determined as the ratio of the respective fractured femur property to the intact femur property.

### μCT evaluation

For μCT analysis, all femurs were scanned in saline by μCT using a Skyscan 1172 high-resolution micro-CT (Skyscan, Kontich, Belgium) with a voxel size of 13.3 μm. The two Skyscan calibration phantoms were included in each scan. Images were reconstructed using the Skyscan software. Mimics 14.11 (Materialise, Ann Arbor, MI, USA) was used to segment the phantoms and fracture calluses.^32^ The linear relationship between the bone mineral content and Hounsfield units (HU)^33,34^ of each scan was calculated using the known density of the two calibration phantoms (0.25 and 0.75 g·cm^−3^) and their segmented HU values.

In Mimics, two transverse slices were used to analyze the bone maturity level of each callus (see Burgers *et al.* ^32^ and Collins *et al.* ^35^ for similar examples). The center of the callus was determined as the location of the small gap between the bones. One slice each was used 0.5 mm proximal and distal to the determined center.^32^ For each slice, four masks were created. The first mask included the total callus cross-sectional area by differentiating the boundary of the callus from the saline using the Mimics thresholding and “3D LiveWire” tools. The second included only the mature area, described by Komatsu *et al.* ^27^ as that with a density greater than 600 mg·cm^−^^3^. The third included only the immature bone area, described by Komatsu *et al.* ^27^ as that with a density of 250–600 mg·cm^−3^. The fourth was the empty (not mineralized) area with a density less than 250 mg·cm^−3^. The second through fourth masks were defined using a Boolean operation with the applicable thresholded density range. The total, mature and immature densities of each region were determined by calculating the mineral content divided by the volume (area times slice thickness) of each mask. Mature bone volume over tissue volume (BV/TV) was calculated as the ratio of the mature area to the total area of the callus. Immature bone volume over tissue volume (IV/TV) was calculated as the ratio of the immature area to the total area of the callus.

### Histological evaluation

For histological analysis, femurs were excised, the needle was removed, and the sample was fixed in 10% neutral buffered formalin for at least 24 h, and decalcified in 10% EDTA, pH 7.5 for 5–8 days at room temperature. The samples were embedded in paraffin along their long axis and sectioned (5 μm). Before staining, slides were deparaffinized and gradually rehydrated though series of ethanol washes. Sections were stained for immunohistochemistry (IHC) and tartrate-resistant acid phosphatase (TRAP). For IHC, in the antigen-retrieval step, slides were incubated in pre-boiled citrate buffer (#005001, Life Technologies, Grand Island, NY, USA) for 10 min. Endogenous peroxidase activity was quenched by 3% hydrogen peroxide for 30 min. The slides were then incubated overnight with primary rabbit anti-β-catenin antibody (#9562, Cell Signaling, Danvers, MA, USA) diluted 1:200. The secondary biotinylated goat anti-rabbit antibody (#BA-1000, Vector Laboratories, Burlingame, CA, USA) diluted 1:200 was used with the Vectastain avidin biotinylated enzyme complex system for visualization. TRAP staining for osteoclasts was performed using a Leukocyte Acid Phosphatase Kit (#387A, Sigma-Aldrich) and counterstained with hematoxylin.

### Statistical analyses

The effect of genotype was assessed statistically. Two-tailed Student's *t*-tests were performed in Excel assuming unequal variance between groups, and *P*<0.05 was considered significant. Average and s.d. are reported.

## Results

**Figure 1:**
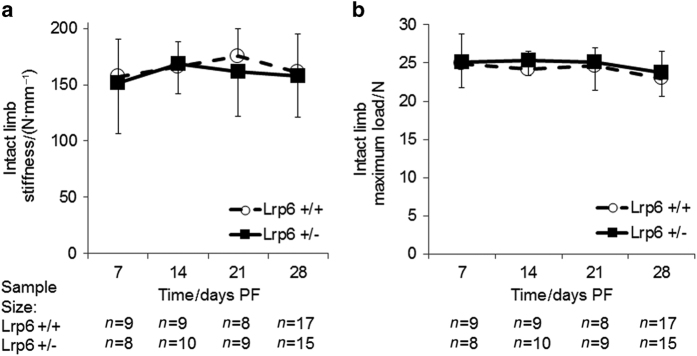
Biomechanical measures of intact bone. There was no statistical difference in (**a**) stiffness and (**b**) maximum load in *Lrp6* ^+/−^ and *Lrp6* ^+/+^ controls at any time point. Error bars indicate one s.d. above the *Lrp6* ^+/+^ group and one s.d. below the *Lrp6* ^+/−^ group.

**Figure 2:**
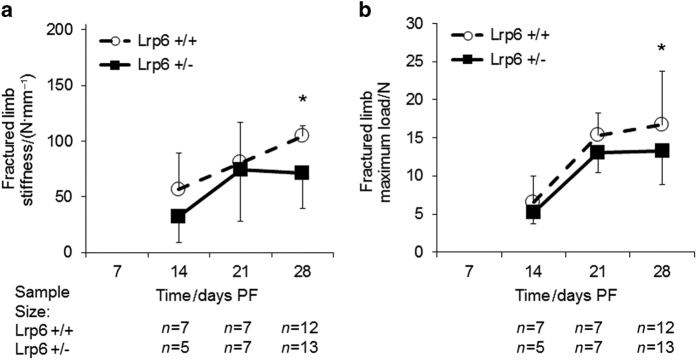
Biomechanical measures of fractured femurs. There was a significant decrease in the (**a**) stiffness and (**b**) maximum load at 28 days post fracture (PF) in the *Lrp6* ^+/−^ group. Error bars indicate one s.d. above the *Lrp6* ^+/+^ group and one s.d. below the *Lrp6* ^+/−^ group (*P<0.05, *Lrp6* ^+/−^ and *Lrp6* ^+/+^ at the time point).

The stiffness and maximum strength (maximum load) were measured in *Lrp6* ^+/−^ and *Lrp6* ^+/+^ femurs intact (Figure 1) and during the process of fracture repair (Figure 2). There was no significant difference in the intact contralateral bones from the *Lrp6* ^+/−^ and *Lrp6* ^+/+^ controls at any time point.

As expected, the stiffness and strength increased throughout healing in both *Lrp6* ^+/−^ and *Lrp6* ^+/+^ groups (Figure 2). Relative to *Lrp6* ^+/+^ mice, *Lrp6* ^+/−^ mice had significantly lower stiffness and maximum strength at 28 days PF. The stiffness and maximum load in the *Lrp6* ^+/−^ group were decreased to 68 and 80% of the *Lrp6* ^+/+^ group, respectively, at day 28 PF.

**Figure 3:**
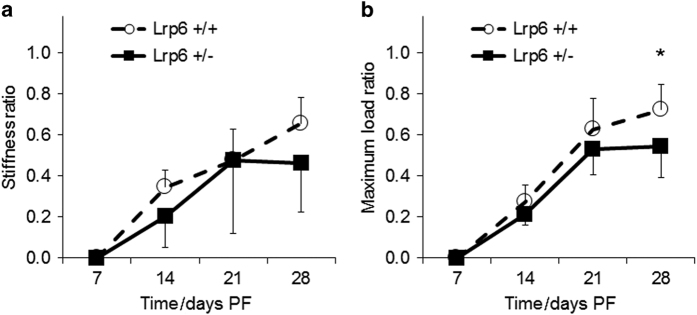
Stiffness and maximum load ratios of fractured to intact femurs. There was no statistical difference in the (**a**) stiffness load ratio at any time point. There was a significant decrease in the (**b**) maximum load ratio at 28 days post fracture (PF) in the *Lrp6* ^+/−^ group. Error bars indicate one s.d. above the *Lrp6* ^+/+^ group and one s.d. below the *Lrp6* ^+/−^ group (*P<0.05, *Lrp6* ^+/−^ and *Lrp6* ^+/+^ at the time point).

At days 14 and 21 PF, the ratio of the biomechanical properties (stiffness and maximum load) of the fractured to intact limb was not significantly different between the *Lrp6* ^+/−^ and *Lrp6* ^+/+^ groups (Figure 3). At day 28 PF, the stiffness ratio was not significant between groups, but the *Lrp6* ^+/−^ had a significantly lower maximum load ratio (75% of the *Lrp6* ^+/+^ group).

**Figure 4:**
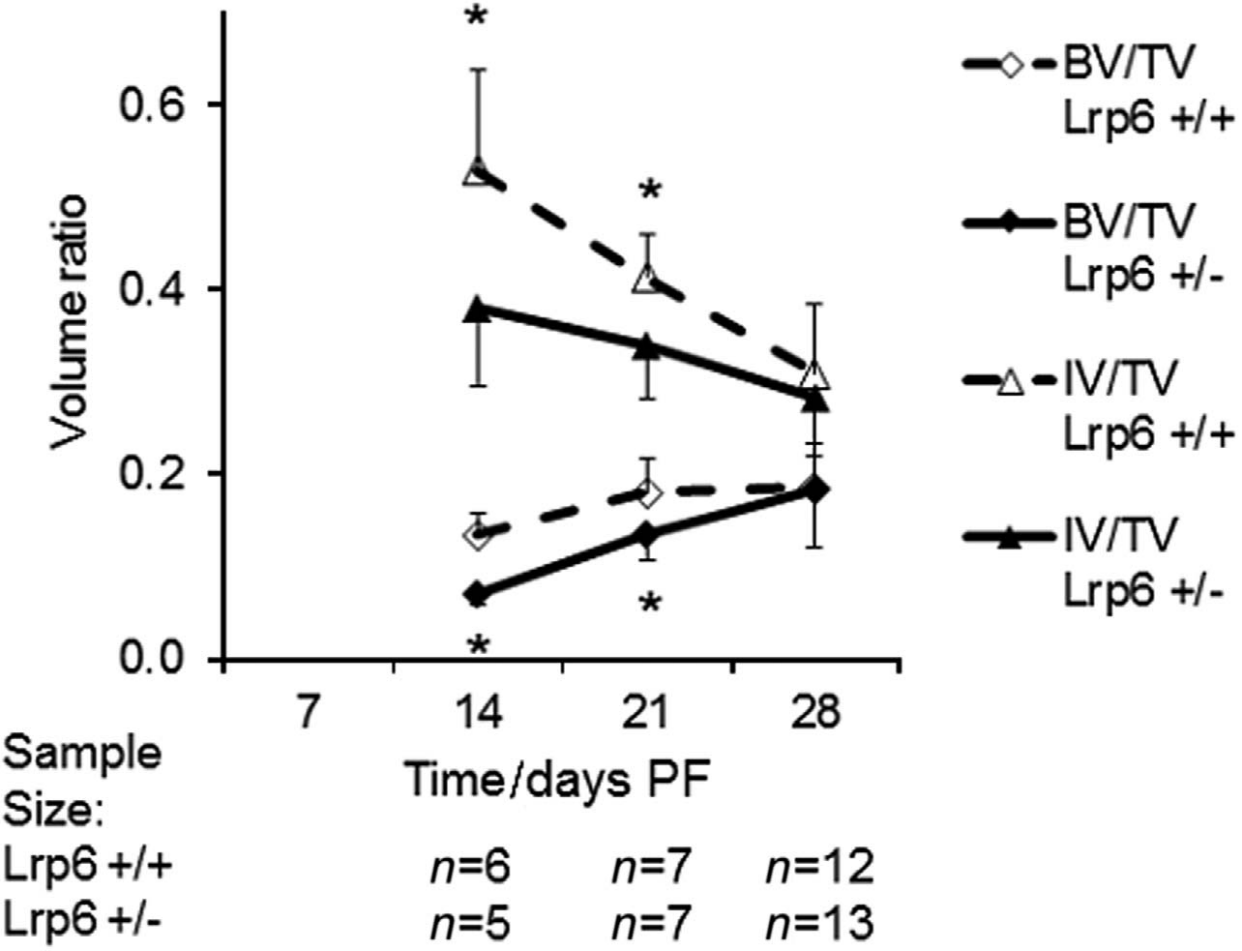
Bone volume over tissue volume (BV/TV) and immature bone volume over tissue volume (IV/TV) ratio for fracture callus. There was a significant decrease in BV/TV and IV/TV at days 14 and 21 post fracture (PF) in the *Lrp6* ^+/−^ group. Error bars indicate one s.d. above the *Lrp6* ^+/+^ group and one s.d. below the *Lrp6* ^+/−^ group (*P<0.05, *Lrp6* ^+/−^ and *Lrp6* ^+/+^ at the time point, and note that IV/TV significance is indicated below the data points).

**Figure 5:**
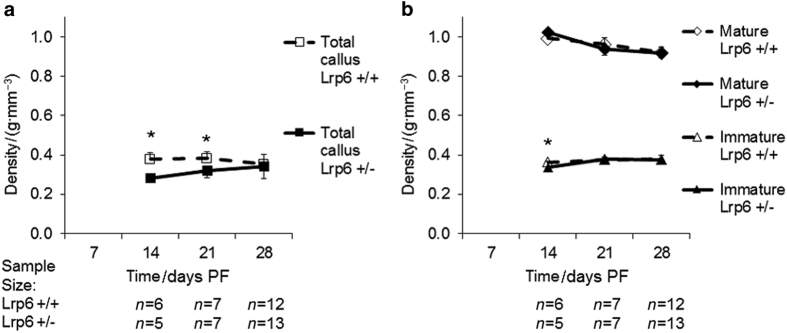
Total callus density, mature callus density, and immature callus density in the fracture callus. There was a significant decrease in (**a**) total callus density at days 14 and 21 post fracture (PF) in the *Lrp6* ^+/−^ group and in the (**b**) immature callus density at day 14 PF. There was no significant difference in the mature callus density at any time point. Error bars indicate one s.d. above the *Lrp6* ^+/+^ group and one s.d. below the *Lrp6* ^+/−^ group (*P<0.05, *Lrp6* ^+/−^ and *Lrp6* ^+/+^ at the time point).

**Figure 6:**
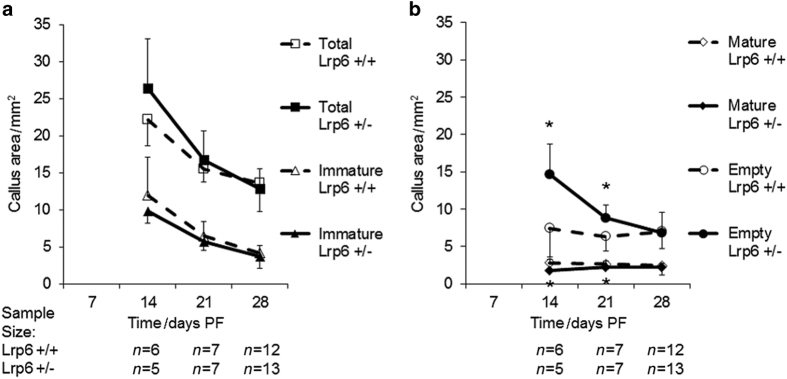
Total callus area, mature callus area, immature callus area, and empty callus area in the fracture callus. There was no statistical difference in the (**a**) total area or immature area at any time point. There was a significant increase in the (**b**) empty area and a significant decrease in the (**b**) mature area at days 14 and 21 post fracture (PF) in the *Lrp6* ^+/−^ group. There was no statistical difference in total area or immature area at any time point. Error bars indicate one s.d. above for the group with the larger area and one s.d. below for the group with the smaller area (*P<0.05, *Lrp6* ^+/−^ and *Lrp6* ^+/+^ at the time point).

To gain further insight into how the heterozygous deletion of *Lrp6* inhibits the late decrease in biomechanical characteristics of the healing bone, the μCT scans of the calluses during healing were examined. At days 14 and 21 PF, the *Lrp6* ^+/−^ group had significantly less callus BV/TV (53% and 74% that of the *Lrp6* ^+/+^ group at days 14 and 21 PF, respectively) and IV/TV (72% and 82%, respectively; Figure 4). The *Lrp6* ^+/−^ group had significantly less total callus density (75% and 83%, respectively) at days 14 and 21 PF and less immature density at day 14 PF (93%); however, there was no significant difference in mature density at any time point (Figure 5). The *Lrp6* ^+/−^ group had significantly less mature area (65% and 82%, respectively) at days 14 and 21 PF and significantly more empty area (198% and 139%, respectively) at days 14 and 21 PF (Figure 6). There was no significant difference in total callus area or immature area at any time point (Figure 6).

**Figure 7:**
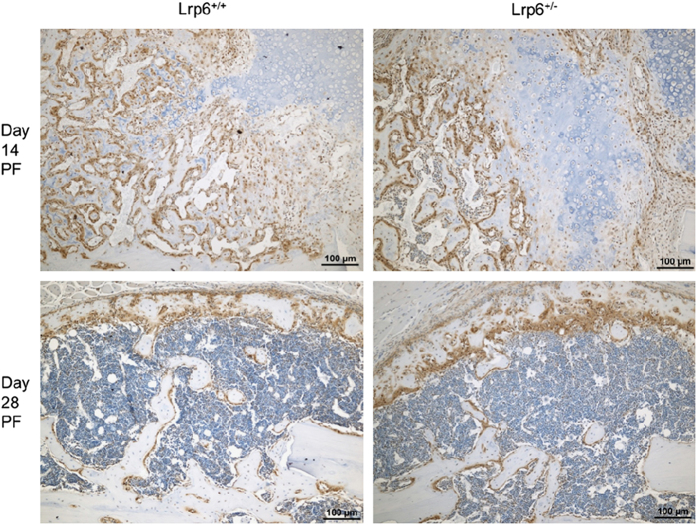
Representative β-catenin staining (for Wnt signaling activity, brown). There is similar β-catenin expression in the *Lrp6* ^+/−^ group (left) compared with the *Lrp6* ^+/+^ controls (right) throughout healing (day 14 post fracture (PF), top; and day 28 PF, bottom, shown here).

**Figure 8:**
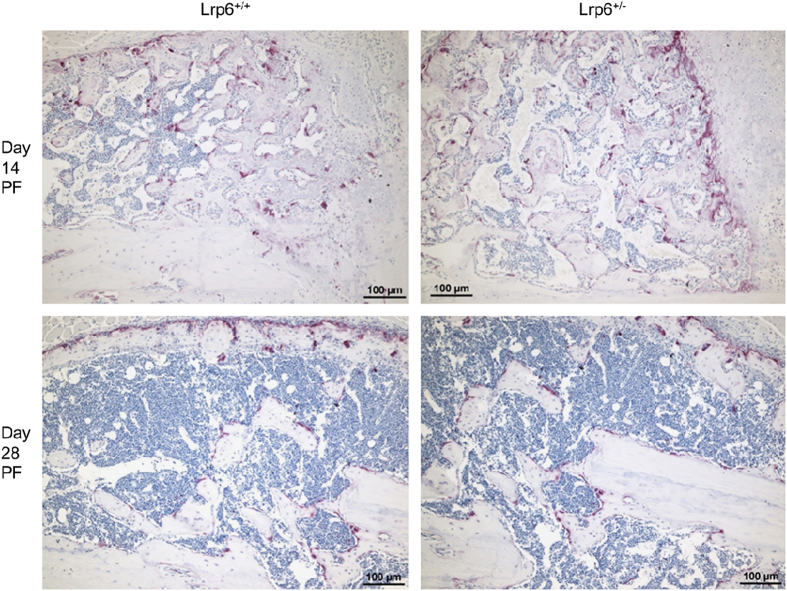
Representative tartrate-resistant acid phosphatase (TRAP) staining (for osteoclasts, purple). There is similar TRAP expression in the *Lrp6* ^+/−^ group (left) compared with the *Lrp6* ^+/+^ controls (right) throughout healing (day 14 post fracture (PF), top; and day 28 PF, bottom, shown here).

To gain further insight into the biological differences within the fracture calluses due to the heterozygous deletion of *Lrp6*, IHC was performed. IHC sections showed no apparent qualitative differences stained for Wnt signaling activity (β-catenin; Figure 7) or osteoclasts (TRAP; Figure 8) at any of the three time points (day 21 PF not shown).

## Discussion

Relative to the *Lrp6* ^+/+^ group, the *Lrp6* ^+/−^ group had no significant difference in the stiffness and strength of intact bones at all time points. In a previous study investigating limb development, the *Lrp6* ^+/−^ group had significantly decreased trabecular BV/TV compared with the *Lrp6* ^+/+^ group. Other bone characteristics were decreased but not significantly so.^17^ The mechanical characteristics are consistent with this result.

Biomechanical analysis also showed that *Lrp6* ^+/−^ mice had significantly reduced stiffness and maximum strength at day 28 PF in the fractured limb. They also had a significantly reduced maximum load ratio of the fractured to intact limb at that time point. This difference later in the healing process demonstrates that the heterozygous deletion of *Lrp6* disrupts at least one stage of endochondral ossification. Fracture healing was also disrupted because of the homozygous deletion of Lrp5^27^ and the osteoblast-specific deletion of β-catenin.^26^ The results reported here along with the previous literature demonstrate the importance of the role of Wnt signaling in the callus osteoblasts and/or chondrocytes during fracture healing.

The μCT analysis showed that at days 14 and 21 PF, the time in which chondrogenesis and endochondral ossification is occurring,^20^ the *Lrp6* ^+/−^ group had significantly less BV/TV, IV/TV, callus density, immature density (only day 14 PF), and mature area compared with the *Lrp6* ^+/+^ group; they also had significantly more empty area. By day 28 PF, there was no significant difference in any of the μCT analyses. The mature bone density decreased over time because of the change of the structure of the callus. Osteoclast activity along the original cortical bone is intense around day 21 PF.^36^ As healing progresses, the interior of the callus also loses its density as the mature bone concentrates on the periosteal surface.^35^ The empty (not mineralized) area in the callus was approximately doubled in the *Lrp6* ^+/−^ group at day 14 PF. This increase in the empty area decreased the total callus density and BV/TV in the *Lrp6* ^+/−^ group. The empty areas that are not mineralized are not completely empty as Figure 7 shows that the callus contains numerous cells in the regions that are not mineralized.

The mature density was likely not different between the groups because there is little gradation in the tissue density of mature bone.^35^ By day 14 PF, mature bone is distributed around the outer portions of the callus,^36^ which is the most biomechanically advantageous location.^8,37^ In intact bone it is common that increased bone density leads to increased stiffness and strength, but this is not necessarily the case for fracture healing. It is most advantageous mechanically for the healing bone to be mineralized farther away from the longitudinal centerline of the bone.^35,37^ For example, during the healing process mineralized callus close to the original cortical bone may be mineralized, but at this location it adds little strength in a bending test. As a result, the μCT measures are useful in measuring some of the mineralization processes of the callus but should be accompanied by mechanical testing because they do not always correlate with stiffness and strength. Because there was no significant difference in the stiffness and maximum load in the callus at days 14 and 21 PF, the decrease in mature area suggests that the mature bone may have been added preferentially toward the inside of the callus.

Although the immature area was not different between the groups, the immature density was decreased at day 14 PF and the IV/TV was decreased at days 14 and 21 PF in the *Lrp6* ^+/−^ group. This suggests that there was some mineralization delay in the immature bone in mineralizing at day 14 PF in the *Lrp6* ^+/−^ group. This is consistent with a recent study reporting that *Lrp6* affects early osteogenic differentiation.^38^ The area and density of the callus was not different between the two groups in the late stages of healing (day 28 PF), but the *Lrp6* ^+/−^ group had significantly decreased stiffness, strength, and maximum load ratio. This, along with the mineralization differences at days 14 and 21 PF, also may indicate that the callus mineralization was more localized toward the inside of the callus where it is not as biomechanically advantageous. To enhance the understanding of the effect of *Lrp6* on fracture healing, future work using finite element analysis could be used to investigate the distribution of mineral within the callus,^39–41^ and nano-indentation studies could be used to investigate tissue-level or location-specific differences within the callus.^42–45^

In a mouse model with a homozygous point mutation in *Lrp6* that results in decreased bone mass, lower bone mass in the *Lrp6* mutant mice was because of increased bone resorption from increased osteoclast activity with no change in bone formation.^46^ In an osteoblast-specific *Lrp6* knockout mouse model, lower bone mass was because of decreased bone formation with no change in osteoclast activity.^38,47^ In this study, the qualitative appearance of the callus did not appear different between the *Lrp6* ^+/−^ and *Lrp6* ^+/+^ groups in the IHC staining. The progression of healing in the cartilaginous callus followed the same pattern as that of bone built during normal endochondral ossification. The qualitative similarities in β-catenin and TRAP staining indicated that there was no marked difference in Wnt signaling activity or osteoclast activity, respectively. It is possible that any difference in biological activity that may have occurred was muted because of only a single deleted allele of *Lrp6*.

The mechanical testing and μCT analyses from this study demonstrate the importance of *Lrp6* in fracture healing and support the results of previous work on the importance of Wnt signaling in fracture healing. As embryonic mice with a homozygous *Lrp6* mutation have severe skeletal deformities,^16^ it is likely that an *Lrp6* homozygous deletion would amplify the fracture healing impairment compared with that presented in this study. It is not possible to investigate fracture healing in a total body homozygous *Lrp6* deletion because these mice are not viable; however, further investigation into the effect of a homozygous deletion could be investigated in a tissue-specific level using conditional knockout mouse models using the Cre-lox system (a bone- or cartilage-specific Cre model^48^ with an *Lrp6* ^fl/fl^ model, for example, refs 18,38,47,49). These conditional knockout models could also be used to further investigate whether *Lrp6* impairs fracture healing due to the chondrogenic response and/or the later osteoblastic response.
